# The Death-Rate of Mothers

**Published:** 1921-06-11

**Authors:** 


### The Death-Rate of Mothers.

Gross as are the hygienic follies still tolerated among us, the principles of hygiene now emerging into recognition have only to be reasonably carried out (Sir Malcolm Morris thinks) to bring us to a state of society in which preventable sickness shall have shrunk to an irreducible minimum. In this connection he pointed out, in a recent address to the Incorporated Institute of Hygiene, that unfortunately the reduced infant mortality in which we can congratulate ourselves has not been attended, during the last twenty-five years, by an appreciable fall in the death-rate of women in child-birth. Every year more than 3,000 women perish in handing on the torch of life to the next generation, while of those who survive the ordeal a substantial proportion rise from childbed to live a life of chronic invalidism. This ought not to be. The agencies by which such a state of things can to a large extent be prevented are already in existence, but evidently they must be called into far more effective operation if this blot upon our civilisation is to be erased. In our view there is a great deal to be hoped for from the new methods of education now in vogue, and the part which the teaching of personal hygiene will increasingly play in the school curriculum. The full effect of such teaching, developed to the highest capacity, will not be realised for another generation. Education, of itself, however, even in close alliance with the organised agencies of public health, will not solve the problems either of infant mortality or the ill-effects of child-birth, so long as every city in the kingdom continues to possess its slum area, or until research and experience have evoked some general plan for dealing with the question of the employment of pregnant women in industry.

